# P01.20. The effect of WIN-34B on cartilage protection and regeneration by chondrogenesis from subchondral bone *in vitro* and *in vivo*

**DOI:** 10.1186/1472-6882-12-S1-P20

**Published:** 2012-06-12

**Authors:** J Huh, Y Park, B Seo, Y Baek, J Lee, D Choi, D Park

**Affiliations:** 1 Kyung Hee University, Seoul, Republic of Korea

## Purpose

WIN-34B is a butanol fraction extract from the mixture of two oriental herbs, the dried Lonicera japonica flowers and the root of Anemarrhena asphodeloides. In previous studies, we indentified that WIN-34B has analgesic, anti-inflammatory, gastroprotective and safety effects. In this study, we measured the major components and investigated the efficacy of WIN-34B on cartilage protection and regeneration through the chondrognesis of mesenchymal progenitor cells of subchondral bone for the treatment of osteoarthritis and development of new medicines.

## Methods

The major chemical composition and quantification of WIN-34B was determined by high performance liquid chromatography. The therapeutic effect of WIN-34B was investigated using a collagenase-induced osteoarthritis (CIA) rabbit model and also by studying chondrogenesis from mesenchymal stem cell of subchondral bone of knee joints.

## Results

In our *in vivo* study using a CIA rabbit model, oral administration of WIN-34B resulted in siginificant reduction of general clinical and histological scores, associated with a significant inhibition of cartilage loss evaluated by the measurement of the proteoglycan and collagen content. The oral administration of WIN-34B against cartilage destruction had more marked effectiveness than that of the specific COX-2 inhibitor, ETCP, Gluco-Hcl in the CIA rabbit model. Immunohistochemistry analysis of this study showed that oral administration of WIN-34B resulted in siginificant increases of CD105 and CD73, typical cell surface antigens known from MSCs. Type II collagen and aggrecan, typical cartilage matrix molecules, were also significantly positive.

## Conclusion

These results suggest that WIN-34B may have shown cartilage protection and cartilage regeneration in a CIA rabbit model through the chondrognesis of mesenchymal progenitor cells of subchondral bone.

